# Structural Biology of the FGF7 Subfamily

**DOI:** 10.3389/fgene.2019.00102

**Published:** 2019-02-12

**Authors:** Allen Zinkle, Moosa Mohammadi

**Affiliations:** Department of Biochemistry and Molecular Pharmacology, New York University Langone Medical Center, New York, NY, United States

**Keywords:** FGF7, FGF10, signaling specificity, crystal structure, threshold model

## Abstract

Mammalian fibroblast growth factor (FGF) signaling is intricately regulated via selective binding interactions between 18 FGF ligands and four FGF receptors (FGFR1–4), three of which (FGFR1–3) are expressed as either epithelial (“b”) or mesenchymal (“c”) splice isoforms. The FGF7 subfamily, consisting of FGF3, FGF7, FGF10, and FGF22, is unique among FGFs in that its members are secreted exclusively by the mesenchyme, and specifically activate the “b” isoforms of FGFR1 (FGFR1b) and FGFR2 (FGFR2b) present in the overlying epithelium. This unidirectional mesenchyme-to-epithelium signaling contributes to the development of essentially all organs, glands, and limbs. Structural analysis has shown that members of the FGF7 subfamily achieve their restricted specificity for FGFR1b/FGFR2b by engaging in specific contacts with two alternatively spliced loop regions in the immunoglobulin-like domain 3 (D3) of these receptors. Weak basal receptor-binding affinity further constrains the FGF7 subfamily’s specificity for FGFR1b/2b. In this review, we elaborate on the structural determinants of FGF7 subfamily receptor-binding specificity, and discuss how affinity differences among the four members for the heparin sulfate (HS) co-receptor contribute to their disparate biological activities.

## Introduction

The fibroblast growth factor (FGF) 7 subfamily is comprised of FGF3, FGF7 (the founding member), FGF10, and FGF22, and constitutes one of five paracrine-acting FGF subfamilies (Itoh and Ornitz, 2004). Members of the FGF7 subfamily are essential for organogenesis and tissue patterning in the embryo, and mediate wound healing and tissue homeostasis in adult mammals (Beenken and Mohammadi, 2009). Specifically, FGF3 is required for inner ear development (Tekin et al., 2007, 2008), FGF7 for the development of the kidney, thymus, and hippocampus (Qiao et al., 1999; Alpdogan et al., 2006; Terauchi et al., 2010; Lee et al., 2012), FGF10 for limb, lung, thyroid, pituitary, lacrimal, and salivary gland (LG and SMG, respectively) development (Bellusci et al., 1997; Min et al., 1998; Xu et al., 1998; Sekine et al., 1999; De Moerlooze et al., 2000; Makarenkova et al., 2000; Hoffman et al., 2002; Izvolsky et al., 2003), and FGF22 for presynaptic neural differentiation (Umemori et al., 2004). Reflecting this functional pleiotropy, aberrant signaling by FGF7 subfamily ligands is responsible for a variety of heritable and acquired human diseases, including congenital deafness (LAMM syndrome) (Tekin et al., 2007, 2008), lacrimo-auriculo-dento-digital (LADD) syndrome (Milunsky et al., 2006), inflammatory bowel disease (Finch et al., 1996), Apert syndrome (AS) (Wilkie et al., 1995; Anderson et al., 1998; Ibrahimi et al., 2001), and prostate cancer (Memarzadeh et al., 2007), among others (Itoh and Ornitz, 2011; Belov and Mohammadi, 2013).

Paracrine FGFs share a core homology region of about 120 amino acids (Beenken and Mohammadi, 2009) that adopt a β-trefoil fold comprised of 12 β-strands (β1 through β12) (Eriksson et al., 1991; Zhu et al., 1991; Osslund et al., 1998; Bellosta et al., 2001; Plotnikov et al., 2001) flanked by N- and C-terminal extensions of variable length and sequence (Mohammadi et al., 2005). These ligands mediate their activities by binding to, dimerizing, and consequently activating cell surface FGF receptors (FGFRs), a family within the single-pass transmembrane receptor tyrosine kinase superfamily. Mammals have four FGFR genes (*FGFR1–4*), each encoding an extracellular portion composed of three Ig-like domains (termed D1–D3) connected by flexible linkers, and an intracellular segment containing a tyrosine kinase domain bounded by flexible N-terminal juxtamembrane and C-terminal tail regions (Mohammadi et al., 2005). Ligand binding requires the D2, D3, and D2–D3 linker regions, whereas the D1 and D1–D2 linker are implicated in receptor autoinhibition (Plotnikov et al., 1999, 2000; Schlessinger et al., 2000; Stauber et al., 2000; Yeh et al., 2003; Olsen et al., 2004, 2006; Liu et al., 2017; Chen et al., 2018). Alternative splicing in the D3 domains of FGFR1–3 generates epithelium- and mesenchyme-specific “b” and “c” isoforms, respectively, with each isoform harboring primary sequence differences in the ligand-binding region in the second half of D3, thus expanding the number of principal FGFRs from four to seven (Orr-Urtreger et al., 1993; Mohammadi et al., 2005).

Paracrine FGFs interact with HS glycosaminoglycans (HSGAG), a mandatory co-receptor/factor in paracrine FGF signaling. HS is a heterogeneously sulfated linear glycan chain of HS proteoglycans, which are ubiquitously expressed either on the cell surface or as soluble components deposited in the extracellular matrix (ECM) (Rapraeger et al., 1991; Yayon et al., 1991; Ornitz et al., 1992; Liu et al., 1996; Perrimon and Bernfield, 2000; Esko and Selleck, 2002). The HS binding site (HBS) of FGFs, housed within the FGF core, is formed by residues from the loop between the β1 and β2 strands as well as the stretch between the β10 and β12 strands (Beenken and Mohammadi, 2009). The HBS regions are rich in basic amino acid residues that engage with sulfate and carboxylate moieties of HS, resulting in avid interaction with HS and sequestration of paracrine FGFs in the ECM. Amino acid variations within the HBS account for the different HS-binding affinities of FGFs across and within paracrine FGF subfamilies. Despite their primary sequence differences, the HBS region between the β10 and β12 strands adopts a common conformation among paracrine FGFs. Nevertheless, in contrast to that of other paracrine FGFs, the conformation of the β10–β11 strand pair HBS region in the FGF7 subfamily is loosely supported by only a single hydrogen bond between the two strands (Yeh et al., 2003).

Heparin sulfate promotes paracrine FGF signaling by orchestrating the formation of a symmetric 2:2 FGF:FGFR dimer on the cell surface. This juxtaposes the intracellular kinase domains in a proximity/orientation necessary for activation loop (A-loop) transphosphorylation, a prerequisite for kinase activation (Plotnikov et al., 1999; Schlessinger et al., 2000; Mohammadi et al., 2005). Following this reaction, additional tyrosine transphosphorylation occurs in the kinase C-terminal tail and juxtamembrane (JM) regions, enabling the activated FGFR to recruit and phosphorylate intracellular signaling molecules (Plotnikov et al., 1999; Mohammadi et al., 2005). In the dimer, FGFRs are located centrally and are bound by both FGFs at the periphery. The dimer interface is mediated by reciprocal contacts between D2 and the FGF ligand from one 1:1 protomer with D2 in the adjoining 1:1 FGF–FGFR protomer. Each HS molecule simultaneously engages the HBS of one FGF and that of the two FGFRs (located in the D2 domain) from both 1:1 protomers (Schlessinger et al., 2000). In doing so, HS enhances the contacts between the FGF and FGFR within each 1:1 protomer in addition to those at the dimer interface, thereby stabilizing the 1:1 complex and buttressing the 2:2 dimer.

FGF–FGFR binding specificity is a key regulator of FGF signaling (Ornitz et al., 1996; Zhang et al., 2006), and is determined by differences in the primary sequences among FGFs and FGFRs, as well as differences in their spatiotemporal expression patterns and HS sulfation motifs. Notably, FGF–FGFR binding specificity establishes bidirectional communication between the epithelium and mesenchyme during development. The FGF7 subfamily is the sole subfamily expressed exclusively in the mesenchyme, and interacts primarily with the “b” isoforms of FGFR2 (FGFR2b) and, to a lesser extent, FGFR1 (FGFR1b) (Mason et al., 1994; Zhang et al., 2006). The remaining four paracrine-acting subfamilies are secreted by epithelial tissues and bind almost exclusively to mesenchyme-specific “c” FGFR isoforms (Ornitz et al., 1996; Zhang et al., 2006; Beenken and Mohammadi, 2009). Because of its tight receptor-binding specificity, the FGF7 subfamily serves as an ideal model for studying the structural determinants of FGF–FGFR binding specificity and function. Indeed, the FGF10-FGFR2b structure – the only FGF7 subfamily FGF–FGFR complex whose atomic structure is currently known – in combination with sequence alignment of the remaining three FGF7 subfamily ligands, has provided major insight into the molecular basis for the entire FGF7 subfamily’s restricted receptor-binding specificity. Furthermore, structural analysis has also shed light on differences among FGF7 subfamily members that explain their non-redundant functions. Specifically, differences in HS-binding affinity suggest that the biological activity of each subfamily member may be governed by distinct thresholds of FGFR dimerization strength, as previously demonstrated in FGF1 (Huang et al., 2017).

## Structural Determinants of FGF7 Subfamily’s Specificity

Based on the 1:1 FGF10:FGFR2b crystal structure (Yeh et al., 2003), the exquisite specificity of the FGF7 subfamily for “b” splice isoform FGFRs is dictated primarily by contacts between ligand and the alternatively spliced regions in the receptor D3 domain. However, the structure also reveals an additional determinant of FGF7 subfamily receptor-binding specificity – namely, a weakened affinity for the D2 domain – which accentuates the subfamily’s reliance on specific contacts with the alternatively spliced D3.

### Specific Contacts With the Alternatively Spliced Regionsin D3

**FIGURE 1:**
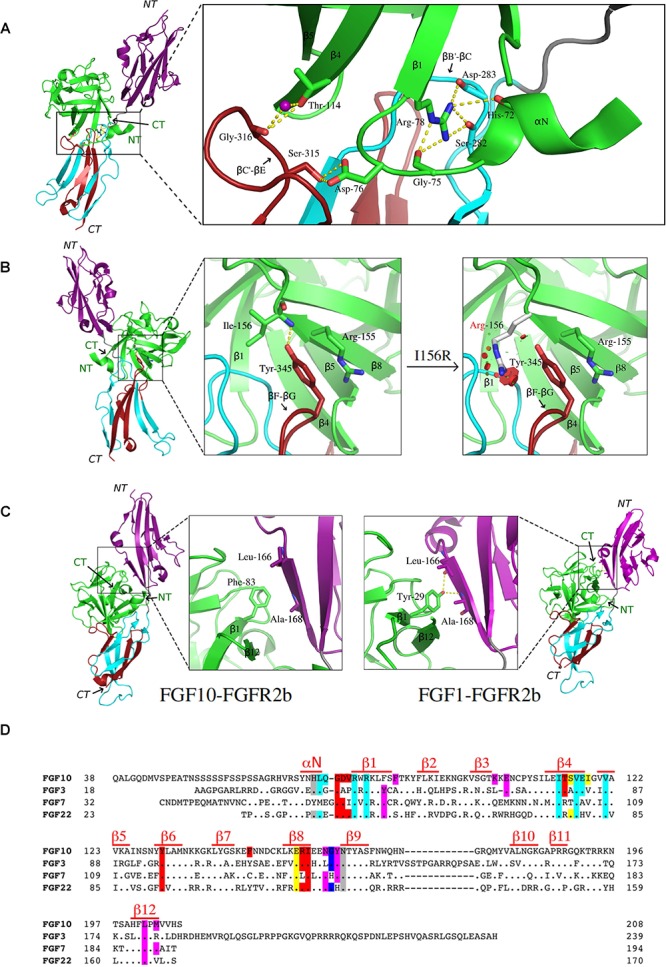
Structural basis for FGF7 subfamily specificity toward “b” isoform FGFRs. **(A)** Left: cartoon representation of the overall structure of the FGF10-FGFR2b complex, with FGF10 in green, D2 of FGFR2b in purple, the constant region of D3 in cyan, the alternatively spliced D3 in red, hydrogen bonds shown as yellow dashes, and the region of interest boxed. N-and C-termini of ligand (in green) and receptor (italics) are labeled NT and CT, respectively. Right: expanded view of FGF10 N-terminal interactions with the FGFR2b D3 domain. Arg-78 of the β1 strand forms three intramolecular hydrogen bonds with Gly-75 and His-72 in the N-terminus, stabilizing it and enabling Asp-76 to form two highly specific hydrogen bonds with Ser-315 in the alternatively spliced βC′-βE loop of D3. Thr-114 of the β4 strand also interacts with the βC′-βE loop via both water-mediated and direct hydrogen bonds with Gly-316. **(B)** Left: overall view of the FGF10-FGFR2b structure, with boxed region of interest shown in expanded forms at right. The first of these (left) shows Arg-155 and Ile-156 of the β8 strand interacting with Tyr-345 on the “b” splice isoform-specific βF-βG loop; the second (right) highlights the LADD mutation (Ile-156 to Arg), which introduces steric clashes with Tyr-345 on the βF-βG loop. Relevant residues and hydrogen bonds are depicted as in **(A)**, with steric clashes illustrated by red circles. **(C)** Comparison of the ligand-D2 interface between FGF10-FGFR2b (left) and FGF1-FGFR2b (right), with each FGF–FGFR complex depicted as a cartoon with the same color scheme as in **(A)**; boxed regions on each complex are expanded to show the ligand β1 strand interacting with D2 of the receptor. Note that in FGF10, a conserved β1 Tyr is replaced with Phe, resulting in a loss of two hydrogen bonds. In **(A–C)**, relevant residues and hydrogen bonds are shown as sticks and yellow dashes, respectively; water molecules appear as purple spheres; oxygen atoms are in red, nitrogen in blue, and carbon in the same color as the molecules to which they belong. FGF10-FGFR2b (PDB ID: 1NUN) (Yeh et al., 2003) and FGF1-FGFR2b (PDB ID: 3OJ2) (Beenken et al., 2012) structures were edited using PyMol (The PyMOL Molecular Graphics System, Version 2.0 Schrödinger, LLC). **(D)** Structure-based sequence alignment of human FGF10, FGF3, FGF7, and FGF22. Dots denote homology with FGF10; dashes denote gaps introduced to optimize sequence alignment. Residues are highlighted according to the FGFR region with which they interact: D2 (purple), D2–D3 linker (gray), constant D3 (cyan), and alternatively spliced D3 (red). Residues which interact with both spliced and non-spliced regions of D3 are highlighted in yellow; those which interact with both the D2 and D2–D3 linker are highlighted in dark blue. Above the sequence, red lines indicate residues comprising secondary structures.

The FGF10-FGFR2b crystal structure shows that most of the FGF10-D3 contacts involve a wide cleft in the membrane-distal end of the D3 domain (Yeh et al., 2003). This cleft is formed between the βB′ strand and the βB′-βC loop located in the constant region (first half of D3) and the βC′-βE loop from the alternatively spliced second half of D3 (Figure 1A). Interactions between Ile-317 on the βC′-βE loop of the receptor and a cluster of hydrophobic residues in FGF10, including Val-116 in the β4 strand, Tyr-131 in the β6 strand, and Phe-146 on the β7-β8 loop, support the formation of the D3 cleft. In doing so, these hydrophobic contacts facilitate hydrogen-bonding interactions between residues from the N-terminus, β1, and β4 strands of FGF10 with residues from both the constant and spliced portions in the D3 cleft (Figure 1A). Most importantly, Asp-76 – conserved in FGF7 and FGF22 – forms two highly specific hydrogen bonds with Ser-315 in the alternatively spliced βC′-βE loop of the receptor. Ser-315 is conserved in FGFR1b, but is replaced by tryptophan and alanine in FGFR3b and FGFR4, respectively, thus explaining the FGF7 subfamily’s particular preference for the “b” isoforms of FGFR1 and FGFR2. Another notable specific receptor-binding residue of FGF10 is Thr-114 on the β4 strand, which engages the alternatively spliced βC′-βE loop of the D3 cleft through both direct and water-mediated hydrogen bonds (Figure 1A). Additionally, Arg-78 in the β1 strand, proceeding Asp-76, makes numerous contacts with the constant βB′-βC loop within the D3 cleft, including three hydrogen bonds with Ser-282 and Asp-283. Crucially, Arg-78 also forms three intramolecular hydrogen bonds with His-72 and Gly-75. These contacts facilitate the overall conformation of the FGF10 N-terminus and indirectly buttress the Asp-76–Ser-315 hydrogen bonds (Figure 1A). FGF10 core residues also augment FGF10-FGFR2b binding specificity by engaging in specific contacts with the alternatively spliced βF-βG loop outside of the D3 cleft. Specifically, Arg-155 and Ile-156 (each in the β8 strand of FGF10) engage in hydrophobic contacts and hydrogen bonding with Tyr-345 in the alternatively spliced βF-βG loop (Figure 1B). Tyr-345 is conserved only in FGFR1b. In FGFR3b, this position is occupied by a phenylalanine, whereas in FGFR1c-3c and FGFR4, the corresponding residue is a serine. These substitutions further limit the specificity of FGF10 for FGFR1b and FGFR2b.

The crystallographically deduced mode of FGF10-FGFR2b specificity has been validated by mutagenesis experiments in FGF10 (Yeh et al., 2003; Wang et al., 2010) and FGF7 (Bottaro et al., 1993; Ron et al., 1993; Gray et al., 1995; Reich-Slotky et al., 1995; Wang et al., 1995; Osslund et al., 1998; Sher et al., 2000, 2003). Specifically, alanine substitutions of Asp-76 or Arg-78 each significantly reduce the biological activity of the respective FGF10 mutants compared to wild-type FGF10, using DNA synthesis as an index of cell proliferation (Yeh et al., 2003). Moreover, replacement of Thr-114 with alanine or arginine decreases FGF10-FGFR2b binding affinity and the mitogenic activity of FGF10 in tracheal epithelial cells (Wang et al., 2010). Conversely, substitutions of His-314 and Ser-315 in the βC′-βE loop in FGFR2b to the corresponding residues in FGFR2c (threonine and alanine, respectively) completely eliminates FGF7 binding (Wang et al., 1995). Also in FGF7, N-terminal truncation at sites upstream of Asp-63 (Asp-76 in FGF10) and Arg-65 (Arg-78 in FGF10), respectively, results in a complete loss of FGF7-induced mitogenic activity in Balb/MK cells (Ron et al., 1993). Additionally, mutations of Asp-63 and Arg-65 to alanine each reduce the binding affinity of FGF7 for FGFR2b, with the latter mutation lowering the mitogenic response of FGF7 in Balb/MK cells by 200-fold (Sher et al., 2003). Replacement of the FGF7 subfamily-conserved Val-103 in the β4 strand of FGF7 (Val-116 in FGF10) (one of the constituents of the aforementioned hydrophobic patch) with glutamic acid also significantly reduces FGF7-FGFR2b binding affinity (Sher et al., 2000). The significance of the Arg-155 (Leu-142 in FGF7)–Tyr-345 interaction has been experimentally validated by data showing that mutating Tyr-345 to serine in FGFR2b significantly reduces receptor activation by FGF7 (Gray et al., 1995), and that replacing Arg-155 with alanine diminishes the ability of FGF10 to promote Balb/MK cell proliferation (Yeh et al., 2003). In FGF7, replacing Leu-142 with alanine results in a three-fold reduction in binding affinity to FGFR2b, as well as a significant loss of mitogenic activity in Balb/MK cells (Sher et al., 2003). Biochemical analysis of a pathogenic mutation in FGF10 lends further support to the importance of these interactions in promoting FGF10-FGFR2/1b signaling. Specifically, mutation of Ile-156 to arginine (I156R) is causative of LADD (Milunsky et al., 2006), a rare genetic disorder characterized by defects in the lacrimal and salivary glands as well as abnormalities in the teeth and distal limbs. Modeling studies show that the I156R mutation introduces steric clashes with residues in the ligand-binding pocket, including Tyr-345 in the alternatively spliced βF-βG loop (Figure 1B), thus explaining the loss-of-function phenotype of this mutation.

### Weak Contacts With Receptor D2 Further Constrain Specificity

FGF7 subfamily receptor-binding specificity is further restricted by its members’ low basal FGFR-binding affinities. Notably, in FGF7, FGF10, and FGF22, a phenylalanine (Phe-83 in FGF10) replaces a highly conserved tyrosine residue in the β1 strand found in all other FGFs (Tyr-29 in FGF1) (Figure 1C). This tyrosine – located at the center of the primarily hydrophobic and largely conserved FGF-D2 interface – makes hydrophobic contacts with residues in the βA′ strand in addition to forming two hydrogen bonds with the FGFR-invariant residues Leu-166 and Ala-168. This conserved pattern of hydrogen bonds fixes the D2 orientation relative to the FGF ligand, accounting for the common D2 disposition among all FGF–FGFR complexes. Replacement of this tyrosine with phenylalanine therefore significantly reduces general FGFR-binding affinity and also causes a ∼20–25° rotation in D2 orientation relative to other FGF–FGFR complexes.

The importance of weak basal FGF–FGFR binding affinity in restricting FGF7 subfamily receptor-binding specificity is underscored by two pathological *FGFR2* mutations, S252W and P253R, each affecting the D2–D3 linker region and causative of AS, a severe form of craniosynostosis (Wilkie et al., 1995; Anderson et al., 1998; Ibrahimi et al., 2001). These gain-of-function mutations create additional non-specific FGF–FGFR contacts that increase basal FGF–FGFR affinity, thereby enabling pathological binding of FGF10 and/or FGF7 to FGFR2c as well as binding of epithelial-expressed ligands such as FGF2, FGF6, and/or FGF9 to FGFR2b (Yu et al., 2000; Ibrahimi et al., 2001). Genetic ablation of *FGF10* in mice harboring AS-causing *FGFR2* mutations reverses some of the skeletal, visceral, and tracheal abnormalities stemming from AS (Hajihosseini et al., 2009; Tiozzo et al., 2009), implying that aberrant FGF10-FGFR2c signaling plays a significant role in AS etiology.

## Molecular Rationale Behind the Non-Redundant Functions of FGF7 Subfamily Members

Gene-knockout studies in mice have shown that despite their shared, restricted specificity for FGFR1b/2b, FGF7 subfamily members have non-overlapping biological functions. For example, while *FGF7*-knockout mice present only subtle developmental abnormalities affecting the kidney, thymus, and hippocampus (Qiao et al., 1999; Alpdogan et al., 2006; Terauchi et al., 2010; Lee et al., 2012), *FGF10*-knockout mice die at birth due to a failure of lung and limb development (Min et al., 1998; Sekine et al., 1999). Structurally, this functional dichotomy between FGF10 and FGF7 can be attributed to the lower HS-binding affinity of FGF7 relative to FGF10 (Igarashi et al., 1998; Luo et al., 2006; Asada et al., 2009). HS-binding affinity differences could impact FGF signaling in one, or both, of the following ways: (1) by changing the extent of ligand diffusion and creating distinct morphogenetic gradients, or (2) by generating distinct thresholds of receptor dimerization and eliciting qualitatively/quantitatively distinct intracellular signals.

A potential role for HS-binding affinity in differentiating the morphogenetic activities of FGF7 and FGF10 can be inferred by data from *ex vivo* epithelial branching model systems in cultured LG and SMG explants (Steinberg et al., 2005; Makarenkova et al., 2009). Due to its relatively low affinity for HS, we postulated that FGF7 would diffuse more readily than FGF10 in an HS-containing matrix (used as surrogate for the ECM) (Makarenkova et al., 2009), thus acting on both the distal and proximal parts of the developing epithelial bud to stimulate branching. On the other hand, the higher HS-binding affinity of FGF10 would limit its range of diffusion such that it can reach only the tip of the epithelial buds, thereby inducing their elongation. To test that these distinct responses are indeed due to different diffusion gradients rather than ligand identity *per se*, we selectively mutated residues at the HBS of FGF10 to the corresponding ones in FGF7, and identified one mutation – Arg-187 in the β11 strand to valine (R187V) – which imparted upon FGF10 a similar range of diffusion as FGF7 (Makarenkova et al., 2009). We then showed that the R187V FGF10 mutant could functionally mimic FGF7 by inducing branching rather than elongation of epithelial buds. It is tempting to speculate that comparable HS-binding affinity differences exist between FGF3 and FGF22 which lead to the generation of FGF7 subfamily ligand-specific diffusion gradients in the ECM, in turn conferring distinct biological activities. However, as the affinity of FGF–HS interactions also dictates the longevity/stability of paracrine FGF–FGFR dimers (Schlessinger et al., 2000), it is plausible that quantitative differences in HS-dependent receptor dimerization may also contribute to the different morphogenetic responses between FGF10 and FGF7. Indeed, the R187V FGF10 mutant is reminiscent of an engineered HS-binding deficient FGF1 mutant which we used to show that the FGF1 mitogenic response could be uncoupled from its metabolic response by reducing FGFR dimer stability (Huang et al., 2017). These data pointed to the existence of a distinct threshold of FGF1-induced receptor dimerization strength required for transitioning from an exclusively metabolic response to a combined metabolic/mitogenic response. Thus, the potential role of HS-dependent FGFR dimerization strength in defining the non-redundancy of the FGF7 subfamily members merits further exploration.

## Conclusion and Future Directions

**FIGURE 2:**
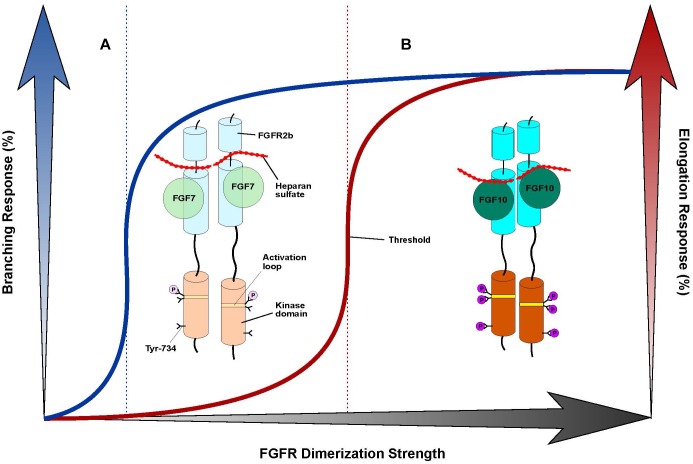
“Threshold model” accounts for differences in branching morphogenesis and FGFR2b tyrosine transphosphorylation between FGF7 and FGF10. Cartoon representation of a “threshold model,” with the FGF7-FGFR2b complex at left **(A)** and the FGF10-FGFR2b complex at right **(B)**. FGF ligands are depicted as different shades of green circles; the FGFR2b ectodomain and kinase domains are shown as cylinders of different shades of cyan and orange, respectively; HS is depicted as a dotted red line; the A-loop region within the kinase domain is shown as a stripe in different shades of yellow; phosphorylated tyrosines are represented as circles colored in different shades of purple. The extent of shading/transparency denotes the strength of ligand-induced FGFR2b dimerization and activation. **(A)** Because of its weak affinity for HS, FGF7 induces comparably weak/transient FGFR dimerization which causes quantitatively less A-loop transphosphorylation/kinase activation such that Tyr-734 is left unphosphorylated; this complex is sufficient to induce branching, but not elongation. **(B)** Owing to its higher affinity for HS, FGF10 forms a more stable FGFR2b dimer that enables greater A-loop tyrosine transphosphorylation and FGFRb activation. Consequently, FGF10 can induce Tyr-734 transphosphorylation and elicit an elongation response. Note that the threshold of FGFR dimerization strength necessary for inducing elongation (depicted as a vertical, red dashed line to the left) is higher than that mediating the branching response (indicated by a vertical, blue dashed line in the center). On the x-axis, a shaded black arrow represents the increasing value of FGF-induced FGFR dimerization strength. On the y-axis, on left, a shaded blue arrow denotes the increasing rate of the branching response, which is correlated with a blue line; on right, a shaded red arrow indicates the increasing rate of the elongation response, which is correlated with a red line.

The FGF7 subfamily is unique among FGFs in that its members exclusively activate and signal through FGFR2b and, to a lesser extent, FGFR1b. The molecular rationale behind this stringent level of receptor-binding specificity stems primarily from specific contacts made between FGF7 subfamily members and the alternatively spliced βC′-βE and βF-βG loop regions in D3, and is further reinforced by weaker D2 interactions (Figure 1C,D). FGF7 subfamily members elicit non-redundant functions, the structural basis of which can be partially attributed to different HS-binding affinities. These affinity differences in turn raise the possibility that the unique functions of each of the FGF7 subfamily ligands may be determined by distinct diffusion gradients through the ECM and/or a “threshold model” of FGFR1b/2b dimerization strength. In this model, different thresholds in FGFR dimer strength/stability translate into quantitatively and qualitatively distinct levels of FGFR activation (that is, tyrosine transphosphorylation). This in turn manifests in different magnitudes of intracellular signals and the recruitment of distinct substrates, culminating in unique cell fates (Zinkle and Mohammadi, 2018). Indeed, previous work has already shown that FGF10 – which binds more tightly than FGF7 to HS – stimulates the recruitment of distinct intracellular substrates due to its unique ability to induce transphosphorylation of a single FGFR2b tyrosine residue, Tyr-734 (Francavilla et al., 2013). Because FGF7-binding cannot induce transphosphorylation of Tyr-734, there may be a certain threshold of FGFR2b dimerization strength that FGF10 (but not FGF7) can reach to induce Tyr-734 phosphorylation (Figure 2). Future studies should try to address the veracity of the threshold model, especially as it relates to the FGF7 subfamily. If validated, this model should prove a reliable guide for functionally converting one FGF7 subfamily member to another, thereby enabling novel tools/strategies for dissecting the roles of individual members of the subfamily during development and in disease pathogenesis.

## Author Contributions

MM contributed to the conception, writing, and revising the manuscript and approved the submitted version. AZ prepared the first draft of the manuscript including the figures and contributed to manuscript revision.

## Conflict of Interest Statement

The authors declare that the research was conducted in the absence of any commercial or financial relationships that could be construed as a potential conflict of interest.
